# Thermo-Optical Studies of Laser Ceramics

**DOI:** 10.3390/ma14143944

**Published:** 2021-07-14

**Authors:** Oleg V. Palashov, Aleksey V. Starobor, Evgeniy A. Perevezentsev, Ilya L. Snetkov, Evgeniy A. Mironov, Alexey I. Yakovlev, Stanislav S. Balabanov, Dmitry A. Permin, Alexander V. Belyaev

**Affiliations:** 1 Federal Research Center, Institute of Applied Physics, Russian Academy of Sciences (IAP RAS), 46 Ul’yanov Street, 603950 Nizhny Novgorod, Russia; astarobor@ipfran.ru (A.V.S.); eperevezentsev@gmail.com (E.A.P.); snetkov@appl.sci-nnov.ru (I.L.S.); miea209@rambler.ru (E.A.M.); alexey.yakovlev@ipfran.ru (A.I.Y.); 2 G.G. Devyatykh Institute of Chemistry of High-Purity Substances, Russian Academy of Sciences, 49 Tropinin Street, 603951 Nizhny Novgorod, Russia; balabanov@ihps-nnov.ru (S.S.B.); permin@ihps-nnov.ru (D.A.P.); belyaev@ihps.nnov.ru (A.V.B.)

**Keywords:** thermal effects, laser ceramics, magnetooptical ceramics, ceramics manufacturing

## Abstract

A cycle of works on manufacturing and studying laser and magnetooptical ceramics with a focus on their thermo-optical characteristics performed by the research team is analyzed. Original results that have not been published before such as measurements of the Verdet constant in the Zr:TAG, Re:MgAl_2_O_4_, and ZnAl_2_O_4_ ceramics are also presented.

## 1. Introduction

A permanent enhancement of the time-average power of continuous and pulse-periodic lasers leads to a significant increase of the parasitic impact of thermal effects on their operation. The thermal effects arising in laser elements, such as thermally induced depolarization and thermal lens, can result not only in a significant deterioration of the radiation quality or loss increase, but also in a reduced stability of optical devices operation up to a complete destruction of them. The fundamental nature of the problem stimulated the development of various methods for its solution, including the search for alternative optical materials possessing optimal laser, spectral and thermo-optical properties. One of the trends nowadays is the creation of laser ceramics.

The principal advantages of ceramics are the possibility of creating large-aperture (like in glass) elements, maintaining of a high (like in a single crystal) thermal conductivity, and the potential of creating ceramics from media that cannot be grown at the present in the form of a single crystal but have unique end user properties facilitated the development of the adequate technologies. Modern technologies enable production of laser elements from ceramics with a sufficient optical quality, a large aperture and a high concentration of dopants. However, this new type of laser materials has features which have no analogues either in glasses or in single crystals and which can negatively manifest themselves in propagation of high time-average power laser radiation through ceramics. Since in a deformed single crystal, the quality of the transmitted radiation depends on the orientation of the crystallographic axes, in ceramics (the grains of which have an arbitrary orientation), the distortion pattern is averaged on grains on ray path. Even cubic crystal ceramics, that may seem to be indistinguishable from similar single crystals, in the presence of mechanical stress (caused, for example, by heating), acquires phase distortions, the inhomogeneity scale of which is comparable to the grain size *l*_g_. This occurs because the rays located at a distance ~*l*_g_ from each other pass through their statistically independent set of grains. These distortions, in turn, lead to the dispersion of thermal depolarization, which degrades the polarization quality of the radiation and the dispersion of thermally induced phase distortions (thermal lens). Thus, both the polarized and the depolarized parts of the radiation that has passed through the thermally loaded ceramic element will have a small-scale modulation with a characteristic size of ~*l*_g_ and an amplitude dependent on the ratio of the sample length to the longitudinal grain size. The theory of thermally induced birefringence in ceramics was constructed and developed in detail in [1,2,3] (both in initially isotropic elements [1,3] and in gyrotropic elements [2]) and was confirmed experimentally in [4,5,6]. In this paper we present results in which the dispersion of thermally induced depolarization and the dispersion of thermally induced phase distortions are negligible.

The use of the ceramics in high-power lasers is currently regarded by the scientific community to be highly promising and the study of the thermal effects in ceramic laser elements is quite relevant. Thermal effects play a significant role both in the generation and amplification of high-power laser radiation in active elements and in the propagation of such radiation through passive optical elements. The calculation, prediction, and selection of methods for reducing and compensating thermal effects is an indispensable part of the development of such laser systems. Therefore, for assessing the potential and specific features of application of new optical materials, it is necessary to determine their material constants.

The absorption of radiation in the optical element bulk leads to an inhomogeneous temperature distribution. As a result, all temperature-dependent optical characteristics (refractive index, thermal conductivity coefficient, Verdet constant, etc.) become inhomogeneously distributed and the internal stresses and, hence, the deformations will lead to the appearance of thermally induced birefringence caused by the photoelastic effect. The inhomogeneous distribution of the refractive index together with the change in geometric dimensions caused by the deformation of the optical elements lead to wave front distortion referred to as the “thermal lens”. The aberrations induced by the thermal lens do not lead to polarizing distortions of the laser radiation, but affect the mode composition of the radiation passing through the optical element [7]. The optical power of the thermal lens D is determined by the ratio [7]:

(1) $$D=\frac{\alpha LP}{4\pi \kappa a^{2}}P_{las},$$

where *α* and *κ* are the coefficients of the medium absorption and thermal conductivity, *P* is the thermo-optical constant [8], L is the length of the optical element, *λ* is the wavelength. *Plas* is the power of incident radiation, and *a* is the laser beam radius.

The depolarization *γ_P_* in a material with a cubic crystal lattice depends on the crystallographic axes orientation relative to the polarization of the incident radiation which is related to the anisotropy of the stress-optic tensor. The transverse structure of the thermal depolarization *γ_P_* with a fourth-order symmetry axis has the shape of the so-called “clover leaf”, more often called the “Maltese cross”. For MAE made of cubic crystal in [001] orientation (in presence of Faraday effect, and angle of rotation of polarization plane *φ* = 45°), minimal value of *γ_P_* can be calculated by the formula [9]:

(2) $$\gamma_{P}=A_{p}p^{2}\min(1,\xi^{2}),$$

where *A_p_* is the coefficient dependent on the laser beam profile (*A_p_* = 0.0139 for a Gaussian beam and *A_p_* = 0.0085 for a flat-top beam and *p* is the normalized laser power:

(3) $$p=\frac{\alpha QLP_{las}}{\lambda \kappa },$$

where *Q* is the thermo-optical constant responsible for depolarization and *ξ* is the optical anisotropy parameter *ξ* (*ξ = π*_44_*/*(*π*_11_ *− π*_12)_*,* where *π_ij_* are elements of stress-optic tensor in Nye notation; for amorphous media *ξ* = 1). The transition to other crystallographic orientations is reduced to the following replacements: ξ → 1, in the case of [111] orientation, *Q* → *Q*(1 + 2*ξ*)/3 [9,10], and for the ceramic element *Q* = *Q*(2 + 3*ξ*)/5 [11] or *Q* = *Q*(53 + 75*ξ*)/128 [2], depending on the grain statistics. For the ceramics, one more term inversely proportional to the number of grains on the laser beam path and to *p*^2^ is added to the expression (2). However, as the characteristic size of the grain in the studied ceramics was small compared to the sample length, this term was neglected.

Thus, the material constants responsible for thermal effects, such as the optical anisotropy parameter *ξ* and the thermo-optical constants *P* and *Q* play an important role in the calculation of thermal effects in lasers with a high average power [8]. For crystals, maximum information about material constants can be obtained from the analysis of thermal effects in the [001] orientation. In this case, the parameter *ξ* and a combination of the material constants *α*_0_|*Q*|/*κ* and *α*_0_*P*/*κ* may be measured separately. If the values of the thermal conductivity κ and the linear absorption coefficient α are known, the values of the thermo-optical characteristics |*Q*| and *P* of the material under study may be determined independently. The value of *P* can be found from Equation (1) using the value of the thermal lens optical power measured in experiment, and the value of |*Q*| from measurements of the minimal thermal depolarization γ. The situation is different for ceramic materials. In this case, only a more complex combination $Q_{eff}=QX$ can be found from the dependence of thermally induced depolarization on laser power, whereas the thermo-optical characteristics cannot be differentiated. However, this information may be sufficient for comparison of the new material with the widely used ones to date.

The team of the authors has been actively involved in the development and investigation of various laser ceramics for many years. A specific feature of our work is the theoretical and experimental study of the thermo-optical properties of ceramics, which, in our opinion, are of key significance for lasers with a high time-average power. That is why the title of this article is “Thermo-optical studies of laser ceramics”, although we also consider other properties of ceramics (magneto-optical, spectral, laser) and do not claim that our research is comprehensive.

Two kinds of laser elements: active elements (AE) of laser heads/amplifiers and magneto-active elements (MAE) of optical isolators (Faraday isolators (FI)) are most strongly subject to thermal self-action due to the inevitable and relatively high absorption of radiation. Two main chapters of the represented work are devoted to them.

## 2. Magneto-Active Ceramics

Faraday isolators are the key elements of laser schemes that are aimed to protect their components from inevitable back reflection that is very dangerous in the work with high-power laser radiation. The main part of the FI is a MAE placed in the magnetic field *H* produced by a permanent magnet system. The Faraday effect leads to the circular birefringence, appearance in the MAE as a result of which the plane of polarization of laser radiation is rotated by an angle *φ* defined by

(4) $$\varphi =V\int_{0}^{L}Hdz,$$

where *V* is the Verdet constant. For proper FI operation the angle *φ* must be equal to 45° and, in an ideal case, the beam is fully reflected by the polarizer on the reverse passage.

Thus, the Verdet constant is one of the most important characteristics of the magneto-active medium as it specifies its length needed to attain the angle *φ* = 45° (*L*_45_). This length, in turn, determines thermally induced distortions of the radiation. For paramagnetic media, the Verdet constant can be increased by cooling the MAE. We studied the spectral and temperature dependences of the Verdet constant for magneto-active ceramics, the obtained results are presented in the work.

Another important FI characteristic is the isolation ratio that is determined by polarization distortions. The isolation ratio *I* is measured in decibels and is defined as

(5) I[dB]=−10lg(γ),

where the radiation depolarization *γ* is understood as:

(6) $$\gamma =P_{d}/P_{las},$$

where *P_d_* is the power of the radiation component polarized orthogonally to the incident radiation by the power *P_las_*. The “cold” depolarization arising in MAEs due to the inhomogeneous and imperfect optical element is small as a rule (10^−5^–10^−4^). The radiation depolarization caused by absorption in optical elements is called “hot” or “thermally induced” depolarization. With increasing laser radiation power, the depolarization increases too and at a certain power it reaches a threshold value for practical application (typically 0.001, or *I* = 30 dB), usually referred to as maximum permissible power *P_max_*.

The inhomogeneous over the radius distribution of the angle of rotation of the plane of polarization caused by the temperature dependence of the Verdet constant leads to a change in the path difference between two circular eigenpolarizations without changing them. The degree of depolarization determined by the dependence of the Verdet constant on the transverse coordinates ($\gamma_{V}$) can be calculated by the expression [12]:

(7) $$\gamma_{V}=B{\left(\frac{\varphi }{\pi /4}\frac{\alpha P_{las}}{\kappa }\frac{1}{T}\right)}^{2},$$

Here, *B* is the numerical coefficient dependent on the laser beam shape that is *B* = 0.00104 for a Gaussian beam, it is assumed that Verdet constant *V* is inversely proportional to temperature *T*: $|V/\frac{dV}{dT}|=T$.

The thermally induced birefringence induced by the photoelastic effect changes the path difference between the eigenpolarizations and the eigenpolarizations themselves that become elliptical at each point. Both effects lead to an inhomogeneous change of the plane of radiation polarization and depolarization.

The contribution of the photoelastic effect to thermally induced depolarization $\gamma_{P}$ is much higher, and the depolarization *γ_V_* due to dependence of the Verdet constant on the transverse coordinates may be neglected [13]. However, in some cases (e.g., in a cryogenic FI) the contribution of the photoelastic effect may be several times weaker. In the work [14] we defined the element length $L^{*}$ at which the magnitudes of depolarizations of both effects become equal:

(8) $$L^{*}=\pi \sqrt{\frac{B}{A}}\left|\frac{\lambda }{QV}\frac{dV}{dT}\right|.$$

For example, for an MAE made of terbium-gallium garnet (TGG) at T = 77 K, $L^{*}$ ≈ 5 mm, while the length of the crystal used in a cryogenic Faraday isolator is *L* ≈ 3 mm. Therefore, $\gamma_{V}$ must be taken into account when estimating thermally induced depolarization, which was confirmed in the work [15], where laser radiation depolarization $\gamma_{V}$ caused by the transverse inhomogeneity of the Verdet constant at MAE heating was revealed and measured for the first time.

If a paramagnetic (for example, TGG) is used as MAEs’ material, the isolation ratio of the device may additionally reduce under the parasitic influence of the effect of paramagnetic MAE magnetization that becomes significant when the magnetic field inhomogeneity inside the element due to magnetization is comparable with the inhomogeneity of the magnetic system field (we showed this effect in the work [16]). Such a situation usually occurs in large-aperture and cryogenic FIs. A new type of a Faraday isolator in which the azimuthally symmetric component of polarization distortions (both under the influence of the effect of paramagnetic MAE magnetization and caused by the transverse inhomogeneity of the Verdet constant at MAE heating) can be made significantly weaker by creating magnetic field inhomogeneity was proposed and demonstrated experimentally in Ref. [12].

We have considered above the features of thermo-optical distortions in an FI. Further, we will analyze the results of our studies of a number of magneto-optical ceramics. We will take as a reference a TGG crystal as it is currently the most widely used magneto-optical material.

### 2.1. TGG Ceramics

TGG ceramics is based on terbium gallium garnet (Tb_3_Ga_5_O_12_). The physical properties of the TGG crystal and TGG ceramics are largely the same: the Verdet constant [17], thermal conductivity, thermo-optical ratio d*n*/d*T*, and thermal expansion coefficient [18,19,20]. We studied experimentally thermally induced distortions in samples of TGG ceramics produced by Konoshima, Japan [20,21,22,23]. We measured the power and temperature dependence of the isolation ratio and optical power of the thermal lens *D* in the 300…90 K range. Also, we developed FIs with different optical schemes, including schemes with compensation of thermally induced depolarization.

**Figure 1:**
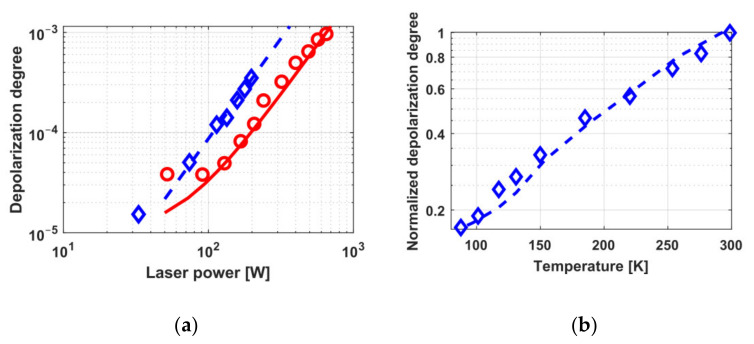
Thermally induced depolarization in TGG ceramics (diamonds—experimental points, dashed curve—theoretical approximation) as a function of laser radiation power at room temperature (**a**) and temperature (**b**). The graph for TGG crystals (circles—experimental points, solid curve—theoretical approximation) with [001] orientation from [24] is given for comparison.

**Figure 2:**
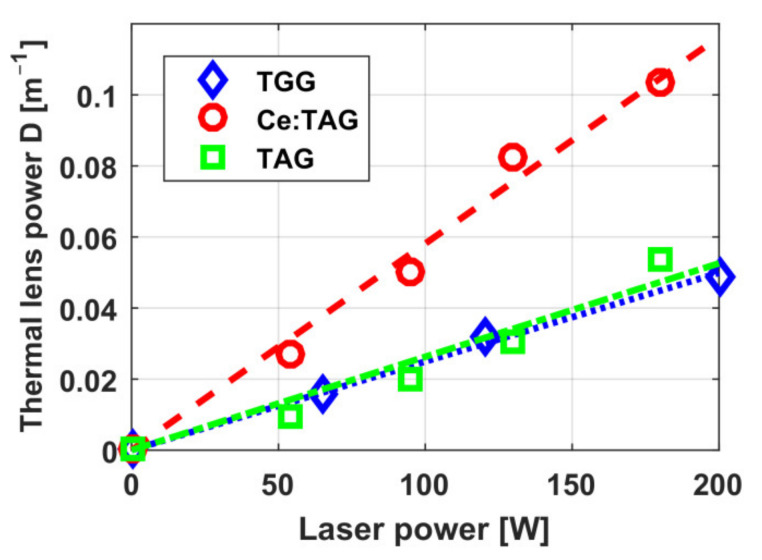
The optical power of thermal lens D as a function of laser radiation power in TGG and TAG-based FIs, Ce(0.1%):TAG.

The dependence of thermally induced depolarization in TGG ceramics on radiation power in a 9.1 mm-long sample and in a 5 mm-long sample with increased absorption is shown in Figure 1 [20,23]. The diameter of all samples was 10 mm. Graphs for TGG crystals with [001] orientation (circles and a solid curve) [24] and with [111] orientation (dashed curve) is given for comparison. Assuming that the thermo-optical characteristics of TGG ceramics are like in the single crystal, we calculated the radiation absorption coefficient to be *α* = 0.0014 cm^−1^, which corresponds to the absorption coefficients of commercial samples of TGG crystals. The thermal lens induced in the samples also corresponds to the value for the TGG single crystal [20]. The dependence of the optical power of the thermal lens *D* on the radiation power from [25] for TGG ceramics, re-calculated for the length *L* = *L*_45_ in a magnetic field of *H* = 2.5 T and a beam having an equal diameter (of 2.4 mm) is plotted in Figure 2. Within the 88–300 K temperature range, the thermally induced depolarization in magnetic field of 1.9 T in a sample of TGG ceramics reduces by more than 6 times on cooling, and *D* by more than 2 times [23].

With the use of a TGG ceramics having length 9 mm as a MAE of the FI with a magnetic system with a maximal magnetic field strength of 2.5 T, the maximal power $P_{max}$ at which the isolation ratio of 30 dB may be attained is 340 W (Figure 1a) [20], which is about twice inferior to that for the TGG crystal with [001] orientation equal to 600 W [24]. Thermally induced depolarization depends on crystal orientation and the optical anisotropy parameter *ξ*. For the case when *ξ* > 0, the optimal orientation is [001] [9], but in view of random grain orientation in ceramics, the properties of the medium are close to the crystal with [111] orientation and it introduces significant depolarization.

A model cryogenic Faraday isolator was also constructed on TGG ceramics with increased absorption (*α* = 0.0056 1/cm) and a length of 5 mm, ensuring an isolation ratio of 28 dB at a power of 450 W. In this case, there arises thermal lens having optical power *D* = 0.6 m^−1^ [23]. Taking into consideration that the optical element of the FI is shortened due to increasing Verdet constant on cooling, the gain in *γ* is a factor of 30, and in *D* a factor of 5. Parameter optimization (reduction of MAE length to 3.5–4 mm and the use of samples with 1.4∙10^−3^ cm^−1^ absorption) of a ceramic sample will enable a *P_max_* increase up to ~1.8 kW.

**Figure 3:**
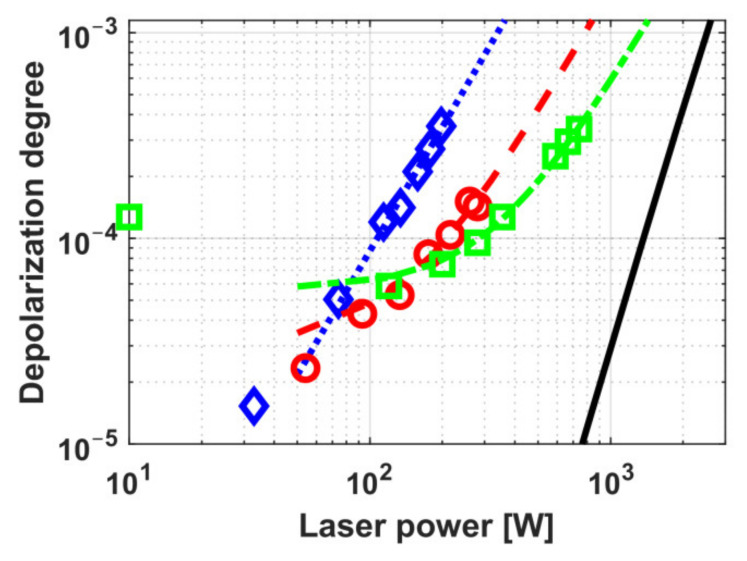
Thermally induced depolarization as a function of laser radiation power in FI based on TGG ceramics without compensation (diamonds—experimental points, dotted curve—theoretical approximation) and with internal (squares and dash-dotted curve) and external (circles and dashed curve) compensation. The solid curve is the theoretical dependence for internal compensation for optical MAE parameters.

The schemes with compensation of thermally induced depolarization in a Faraday isolator using ceramic MAEs were first implemented on TGG ceramics in the works [21,22]. Two schemes were considered: with internal [26] and external [27] compensation. The idea underlying these schemes implies the use of two optical elements, in which there arises thermally induced depolarization, and a polarization rotator between them that reverses the sign of the birefringence, thus partially compensating the thermally induced depolarization. In the scheme with internal compensation, the MAE is divided into two identical elements between which a reciprocal polarization rotator is placed. In the case of external compensation, besides a conventional MAE, a reciprocal rotator and an additional optical element (AOE) that does not rotate the polarization plane are used. The principal advantage of this scheme is a possibility to modify traditional FIs avoiding changes in their magnetic system and MAE and to use as an AOE a different, not necessarily magneto-active material [27]. Internal compensation permitted reaching the isolation ratio up to 35 dB at a radiation power of 740 W [22]. External compensation enabled an increase in the isolation ratio at a power of 340 W from 30 to 38 dB [21]. According to the estimates, *P_max_* > 2 kW in both schemes using optimized MAE length. The dependence of the depolarization degree on the radiation power for both compensation variants is presented in Figure 3.

Thus, TGG ceramics is comparable to TGG crystal as a medium for FIs for lasers with high average power, including the cases of nitrogen temperatures. At the same time, it allows producing large-aperture Faraday isolators operating with high-power laser radiation, which opens up new opportunities for creating lasers with high time-average and peak power.

### 2.2. Re:TAG Ceramics

One of the materials promising for use in FIs is terbium aluminium garnet Tb_3_Al_5_O_12_ (TAG) that surpasses TGG in the value of the Verdet constant by ~37% [28,29,30]. However, it is very difficult to grow TAG single crystals with acceptable aperture because of their incongruent melting nature and unstable TAG phase in the Tb_2_O_3_-Al_2_O_3_ system [31]. Several research teams have been developing various methods for growing TAG crystals, but their optical aperture still does not exceed several millimeters [30,32]. Therefore, we believe that an optimal way to obtain such a medium is to produce TAG ceramics. The first results in manufacturing TAG ceramics samples, as well as the study of the optical quality, microstructure, magneto-optical property, and thermal conductivity were reported in [33]. The technology of ceramics allows producing optical elements made of TAG ceramics of arbitrary size, up to tens of centimeters in cubic garnet phase, avoiding problems related to crystal phase instability in melt [34]. Currently, several teams manufacture high-quality TAG ceramics employing different methods [35,36,37]. In the experiments described in this section, the ceramics produced by the Chinese institutes SIOM (Shanghai, China) [37] and SICCAS (Shanghai, China) [35] was used.

The technology of growing ceramics allows introducing into its composition both sintering additives, that increase the transparency of the medium, and additional ions, that can affect the Verdet constant. We investigated TAG ceramic samples doped with Ce, Pr, Ho, Si, Ti, and Zr elements with a concentration of up to 2 at.%. According to the measurements, the value of the Verdet constant in the studied ceramic samples does not depend on the dopant type and concentration to an accuracy of the experimental error. However, in the literature there is a wide scatter in the values of the measured Verdet constant both in pure and doped TAG, which in some cases leads to contradictory conclusions about the dependence or independence of the Verdet constant on dopants. This is especially true if the effect is rather week (~10–15%) or the doping is low (1–5 at.%). The literature sources and possible causes of the significant scatter in the Verdet constant values were analyzed in the work [38], including

- the temperature dependence of V (~1/T) in paramagnetic materials, to which TAG belongs;
- the accuracy of determining the wavelength of radiation sources;
- the accuracy of determining the angle of rotation of the polarization plane;
- the accuracy of determining the magnitude/integral of the magnetic field and the position of the sample in it;
- a high unaccounted concentration of sintering additives and impurities that may exceed the concentration (or influence) of the declared dopants, etc.

**Figure 4:**
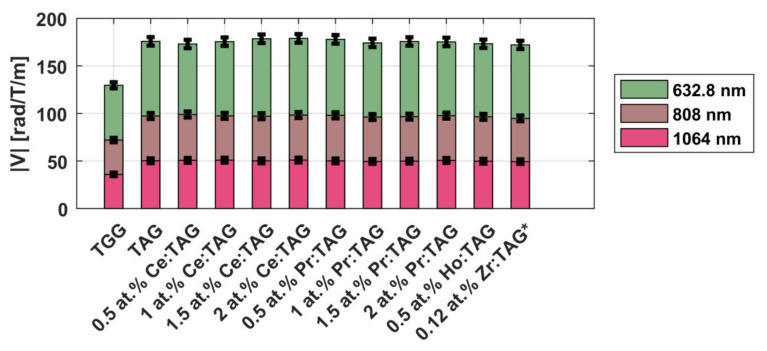
Verdet constant of the TGG and Re:TAG ceramics samples at the wavelengths 1064, 808 and 633 nm [38]. *—unpublished results.

**Figure 5:**
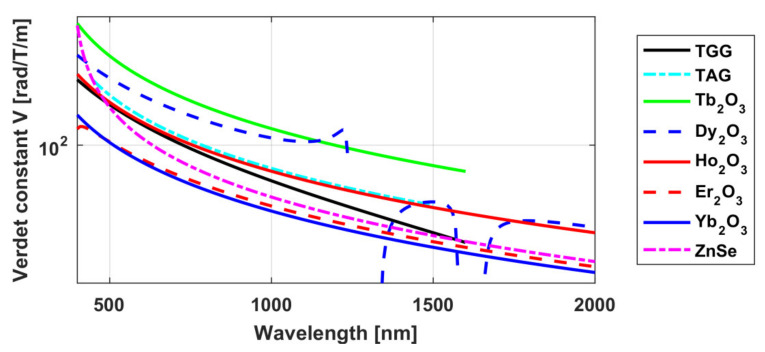
Dispersion trends of the Verdet constant of TGG [39], Tb_2_O_3_ [39,40], TAG [38,41], Dy_2_O_3_ [42], Ho_2_O_3_ [43,44], Er_2_O_3_ [45], Yb_2_O_3_ [46], ZnSe [47], and Y_2_O_3_ [42]).

The spectral dependence *V*(*λ*) of TAG ceramics may be approximated for all samples to an accuracy better than 5% in the form:

(9) $$V(\lambda )=\frac{C}{\lambda^{2}-\lambda_{0}^{2}},$$

where the values of the coefficients *C* and *λ*_0_ for the studied ceramics are *C* = 5.33 × 10^7^ (rad × nm^2^)/(T × m) and *λ*_0_ = 290 nm. The experimental values for the Verdet constant at the wavelengths 633, 808 and 1064 nm are given in Figure 4. The spectral dependence of the Verdet constant in TAG compared to TGG ceramics and other media is presented in Figure 5.

The addition of sintering additives with absorption bands in the visible and near-IR regions of the spectrum ensures a significant increase of absorption in TAG ceramics. Measurements of thermally induced effects [25,41,48,49] show that the smallest depolarization degree is introduced by pure TAG and TAG doped with silicon and cerium ions that have no absorption bands in this region, despite the fact that the highest transparency is shown by the samples doped with zirconium oxide [50].

**Figure 6:**
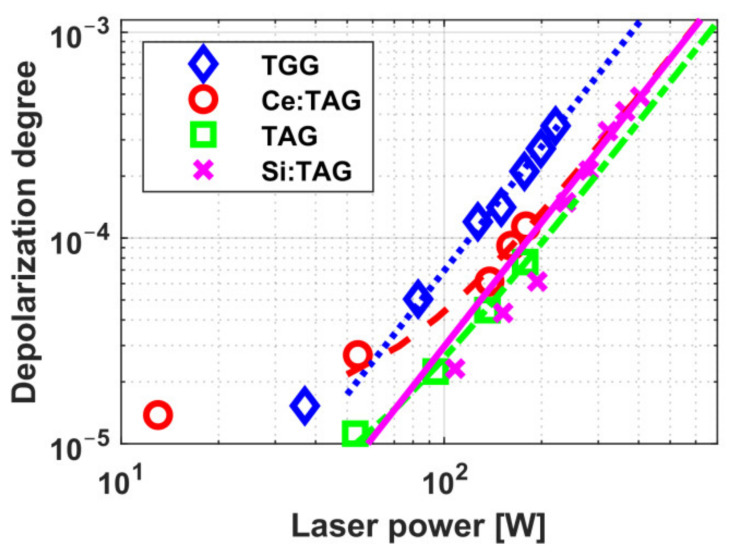
Depolarization degree versus laser radiation power in FI based on TGG, TAG, Si:TAG, and Ce:TAG ceramics.

The thermally induced depolarization as a function of the radiation power for best samples of these ceramics of *L*_45_ in a magnetic system with a field strength of 2.5 T is plotted in Figure 6. It is clear from the graph that *P_max_* of the TAG ceramics is ~1.5 times higher than of the TGG ceramics.

The dependences of the optical power of thermal lens *D* on the radiation power reported in [25,48,49] for TAG and Ce(0.1%):TAG re-calculated for the length *L* = *L*_45_ and beam diameter of 2.4 mm are shown in Figure 2. The value of *D* is approximately equal for TAG and TGG, but is about 2 times higher for Ce:TAG, as the absorption of the sample with the content of cerium of 0.1% is higher than of the sample with 0.05%.

With the use of magneto-optical ceramics we have developed a FI with an isolation ratio of 38 dB for a radiation power of 300 W and a focal length of the thermal lens of about 8 m [48]. The maximum power *P_max_* was estimated to be >700 W. For an FI based on Ce:TAG we took a sample with a cerium content of 0.1%, i.e., with the absorption being higher than in the case of an optimal cerium content of 0.05%, and obtained only 31 dB at a radiation power of 300 W. The use of the internal scheme of compensation of thermally induced depolarization allowed us to increase the isolation ratio up to 39 dB for the same power [49] and to obtain *P_max_* >2 kW.

**Figure 7:**
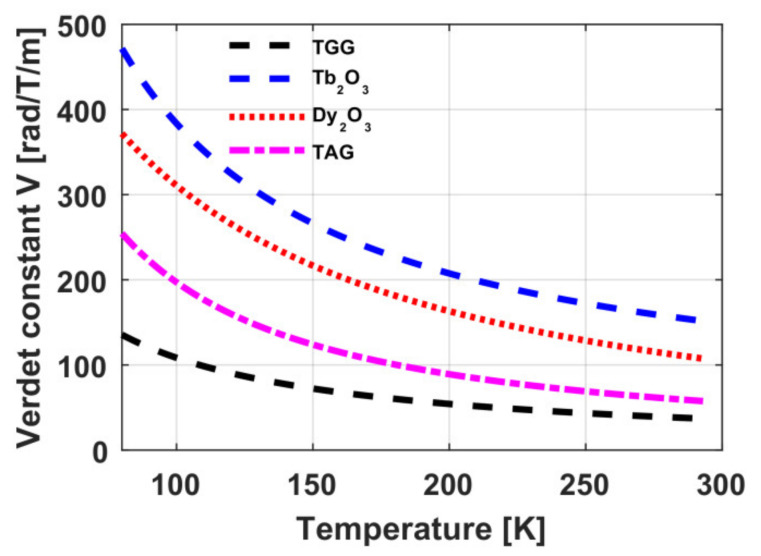
Verdet constant as a function of temperature at wavelength of 1064 nm: TGG [18,23], TAG [51], Tb_2_O_3_ [53], Dy_2_O_3_ [54].

Cryogenic cooling enables an additional reduction of thermally induced distortions and shortening of MAE length due to an increased Verdet constant. On cooling, the Verdet constant *V* in TAG ceramics increases faster than 1/*T*, and at 89 K it amounts to V(1064 nm) = 221 rad/T/m [51]. The temperature dependence of *V* is plotted in Figure 7. The thermally induced depolarization in a TAG ceramics sample reduces by more than 5 times, and the optical power of the thermal lens by more than 2 times as a result of cooling from 295 to 79 K. In view of the increase of the Verdet constant at cooling to 79 K, in a magnetic cell with a field strength of 1.9 T it is sufficient to use an element with a length of 2.3 mm to obtain, according to the estimates, *P_max_* > 3 kW. However, due to the high scattering in the sample at cooling, the depolarization increases and at cooling to 150 K, *P_max_* = 0.5 kW [51,52].

Note that in the majority of our TAG ceramics samples we observed strong additional heating caused by scattering in the samples, which demanded active cooling of the MAE. Thus, being superior to TGG in the Verdet constant, TAG ceramics, after further improvement of its optical properties, can be successfully used in isolators for lasers with high average power.

### 2.3. Sesquioxide Ceramics

The prospects of using sesquioxide ceramics thanks to their thermo-optical properties arose the interest of researcher more than 50 years ago [55]. We pay great attention to manufacturing [46,56,57,58,59,60], as well as to studying [39,40,42,43,54,57] magneto-optical and thermo-optical properties of the sesquioxide ceramics Y_2_O_3_, Tb_2_O_3_, Dy_2_O_3_, Ho_2_O_3_, Er_2_O_3_, and Yb_2_O_3_, that will be considered in this section.

#### 2.3.1. Production Technique

To date, many methods have been developed for both the synthesis of initial rare-earth oxide nanopowders and methods for their subsequent consolidation into optical-quality ceramics. In our works, we use the method of self-propagating high-temperature synthesis (SHS) to obtain nanopowders containing matrix elements uniformly distributed over the volume, sintering and auxiliary additives (LiF, ZrO_2_, and rare earth oxides other than the main matrix oxide) [58,60]. SHS consists in the synthesis of a precursor containing nitrates of the corresponding metals and organic fuel—acetic or aminoacetic acids, followed by thermal initiation of its combustion. In the course of the reaction, the precursor melts and it is foamed by the evolved gases. The walls of this foam are one or tens of nanometers thick and consist of metal oxides. Fast combustion (a few seconds) and a significant volume of evolved gases prevent the particles from sintering with each other. Therefore, the foam crumbles to submicron particles even on insignificant mechanical impact (pressing, grinding, ultrasonic dispersion, etc.). Depending on the initial components and the features of SHS, the specific surface area of oxide powders of rare-earth materials (REE) varies from 30 to 100 m^2^/g. The advantages of the method are the simplicity and technological flexibility of the process, which allows synthesizing mixed compositions with a homogeneous distribution of components, a small number of stages limiting the contamination of materials, and a high sinterability of the resulting powders. The main drawbacks of the method are the presence of inhomogeneous combustion areas (for example, near the walls), where rigid agglomerates can form, as well as a predominantly lamellar shape of the particles and a low bulk density of the powders, which hinder the formation of compacts with a homogeneous pore structure. The latter, among other things, imposes restrictions on the methods of their subsequent consolidation. So, without additional processing of powders (annealing and grinding), the achievable maximal thickness of laser-quality ceramics obtained by free vacuum sintering does not exceed 1–2 mm. Thicker specimens can be obtained by hot pressing, in which the applied uniaxial pressure provides additional driving force to the consolidation.

The sinterability of the individual oxides is usually insufficient for producing laser-quality ceramics without hot isostatic pressing and is a nontrivial task, even with its application. Therefore, at free vacuum sintering, a sintering additive La_2_O_3_ [45,58,61] and in some cases Y_2_O_3_ is used, when the solubility of lanthanum oxide is insufficient for a single-phase ceramics to be formed (for example, in Dy_2_O_3_ [42,56]). These additives are isovalent to REE oxides and, except for some decrease in thermal conductivity, have do not negatively affect the characteristics of ceramics. At hot pressing, lithium fluoride is the most effective sintering additive [40]. It significantly activates the diffusion processes and is almost completely removed from the ceramics at the stage of open porosity. Thus, it has no impact on the final characteristics of the ceramics, except for some increase in the average grain size.

After sintering, an oxygen deficiency is usually formed in the ceramics, which gives them a dark shade and markedly reduces the transmittance in the visible and near-IR wavelength ranges. Therefore, before optical processing, the ceramics are annealed in air at temperatures of about 900 °C for 5 h. The only exception is the ceramics based on terbium oxide, which is oxidized to Tb_4_O_7_ in air [59] and becomes opaque in the entire optical range.

#### 2.3.2. Results of the Studies

Samples of magneto-active ceramics were studied in detail in the following works: Tb_2_O_3_ [39,40,53], Dy_2_O_3_ [42,54,62], Ho_2_O_3_ [44,45], Er_2_O_3_ [57], Yb_2_O_3_ [46], and Y_2_O_3_ [42]. The research [44] was made in collaboration with the Chinese colleagues of the Shanghai Institute of Ceramics. The theoretical dependences of the Verdet constant on wavelength obtained by approximating the experimental data of the above-mentioned works using the expression (9) in comparison with the widely used TGG material are presented in Figure 5 [63]. The approximation of the Verdet constant of yttria ceramics was obtained based on the data from [42] with the approximation coefficients *C* = 2.07 × 10^6^ (rad × nm^2^)/(T × m) and *λ*_0_ = 232 nm from the expression (9). It is worth noting that these approximations do not describe the behavior of the Verdet constant in the regions with absorption, but give its good description in the wavelength ranges in which experiments were conducted in the above works.

The Verdet constant of undoped ceramics Y_2_O_3_ has a ~20 times lower absolute value than TGG [42]. The values of the Verdet constant of Yb_2_O_3_ and Er_2_O_3_ are close and are ~2 times less than of TGG in the range of its operation (up to ~1.4 µm). A distinguishing feature of Yb_2_O_3_ and Er_2_O_3_ ceramics is the absence of absorption lines in the 1.1–6 µm and 1.7–7 µm ranges, respectively, so their application as a magneto-active material is promising only in the small range of 1.7–1.9 µm.

**Figure 8:**
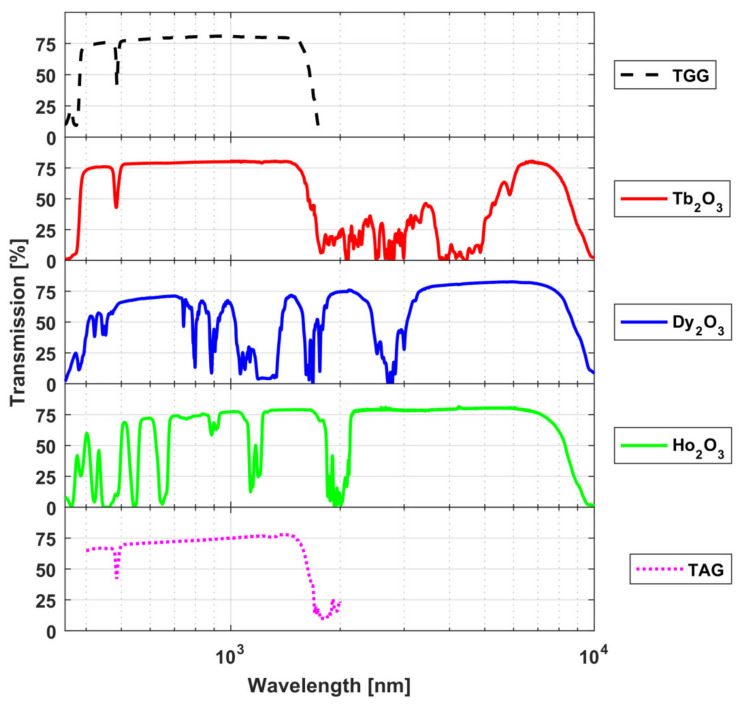
Recommended ranges of transmission spectra: TGG [39], Tb_2_O_3_ [39,40], Dy_2_O_3_ [42], Ho_2_O_3_ [43,44], TAG.

As the other ceramics (Tb_2_O_3_, Dy_2_O_3_, Ho_2_O_3_) have a much larger Verdet constant and surpass even TGG, they are interesting as potential candidates throughout their transparency ranges/windows. Tb_2_O_3_ ceramics, like all Tb-containing materials have a multiple absorption bands, starting at about 1.3–1.4 µm; Dy_2_O_3_ has several absorption lines in the 1–1.9 µm and 2.3–3.5 µm ranges, and Ho_2_O_3_ in the 1.07–1.3 µm and 1.65–2.2 µm ranges. The most attractive recommended ranges of the considered ceramics are clearly seen in the transmission spectra in Figure 8. In the range up to ~1.4 µm, the most preferable materials are Tb_2_O_3_ and TAG (partly Dy_2_O_3_); in the 1.5–2.1 µm—Dy_2_O_3_ or Ho_2_O_3_ (at shorter or longer wavelengths *λ*, respectively); in the 2.1–3.2 µm range Ho_2_O_3_ is the leader (due to transmittance); in the ~3.2–6 µm range Dy_2_O_3_ is preferable; and in the 6–8 µm interval Tb_2_O_3_ takes the leadership again (thanks to the large Verdet constant).

Cryogenic cooling is a possible way to increase the Verdet constant of MAE made of a paramagnetic material [64], which can decrease the element length and, hence, increase the magnitude of thermally induced distortions. The theoretical dependences of the Verdet constant on temperature for Tb_2_O_3_ and Dy_2_O_3_ ceramics obtained in our studies [53,54] are given in Figure 7. According to our results, on cooling, the Verdet constant increases in proportion to the inverse temperature.

### 2.4. Zinc Selenide

Zinc selenide (ZnSe) obtained by chemical vapor deposition (CVD) has found the widest application in power optics [65]. Its distinguishing feature is the optical transmittance close to the theoretical limit, high chemical and phase purity, weak mechanical stresses and small deviations from the stoichiometry of the components, as well as the possibility of manufacturing large-size elements. The CVD process runs in a graphite flow reactor with a square or rectangular cross section. Zinc and hydrogen selenide vapors with argon carrier gas are fed into the reactor, where their chemical interaction takes place. ZnSe is deposited on graphite substrates, and the resulting hydrogen is continuously removed with argon by a vacuum pump. Typical temperatures in the reactor are 700–750 °C, the absolute pressure is about 0.5–5 kPa, the deposition rate is 2–2.5 mm/day, the area of one graphite substrate can reach 2 m^2^ with a layer thickness of tens of mm. The resulting ZnSe sheets are removed from the substrate, cut into elements, and optically processed.

During the process, the initial zinc and selenium undergo a number of physical and chemical transformations that ensure effective purification to give the purity of CVD-ZnSe of 4 N or more. The synthesis temperature is less than half the melting point of the material. This makes it possible to reduce the intake of impurities from the materials of the equipment, maintain a relatively low average grain size of 20–80 µm, significantly reduce the solubility of superstoichiometric zinc or selenium in the matrix, and, hence, reduce the probability of secondary phase formation when ZnSe is cooled to room temperature, as well as reduce mechanical stresses due to the absence of phase transitions during cooling. The disadvantages of the CVD method are its technological complexity, low material growth rate, high toxicity and explosion-fire hazard of intermediate compounds, and the yield of the laser-quality target material rarely exceeding 25% in terms of the initial components.

**Figure 9:**
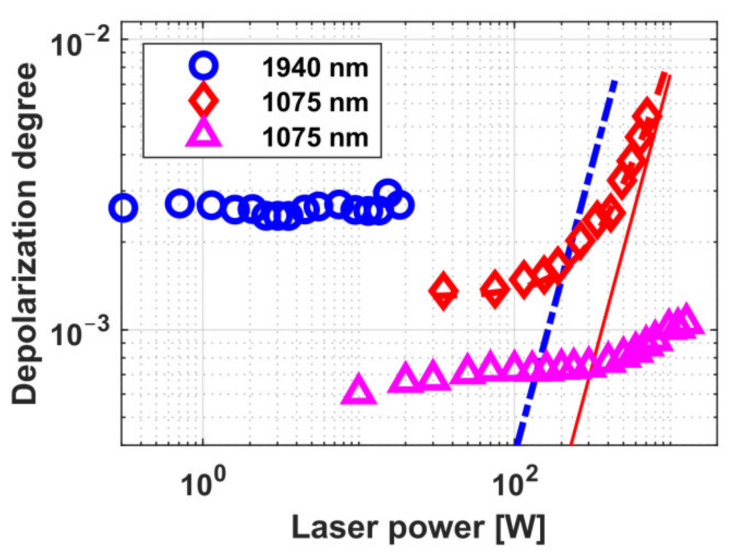
Power dependence of radiation depolarization in FIs based on CVD-ZnSe elements for a wavelength of 1940 nm (circles—experiment, dash-dot line—calculation [47]) and for a wavelength of 1075 nm (diamonds—experiment, dashed curve—calculation [47], triangles—experiment [47,66].

We measured the dispersion dependence of the Verdet constant *V*(*λ*) of polycrystalline CVD-ZnSe at room temperature in the range of 532–1940 nm in the work [47]. The diamagnetic rotation of the polarization plane was observed, which was opposite to that of paramagnetic crystals (i.e., the sign of the Verdet constant of ZnSe was positive). The experimental dependence is well approximated by the expression (9). For *C* = 2.55∙10^7^ rad∙nm^2^/(T∙m) and *λ*_0_ = 380 nm, the approximation accuracy was 9%. The graph of the approximated dependence is shown in Figure 5. At a wavelength of 1064 nm, the Verdet constant of ZnSe was 28.3 rad/T/m, which is a little lower than that of TGG (~22%). Therefore, it might seem that this material is of the greatest interest for laser radiation at wavelengths over ~1.5 µm, where TGG has high absorption and stops working. The Verdet constant at a wavelength of 1940 nm was 7.7 rad/T/m, which is a fairly high value. The combination with the optical transparency of ZnSe in this range made it possible to implement the corresponding FI. Thermally induced depolarization was not observed experimentally up to 20 W and according to the estimates, $P_{max}$ was >150 W [47]. The dependence of the polarization of the transmitted radiation on its power is shown in the graph in Figure 9 (circles and dash-dot line). The FI for *λ* = 1075 nm was also developed and investigated, with $P_{max}$ ~350 W (Figure 9, diamonds and dashed curve).

Note that we are just starting to work with chalcogenides and, believe that they are very interesting and promising materials for high-power FIs. The CVD-ZnSe samples under study were obtained in processes with different accuracy of controlling the reaction parameters. An isolator for the radiation wavelength of 1075 nm was also explored on the basis of a sample synthesized in the process with a more accurate maintenance of the stoichiometric ratio of the reaction components [66]. No thermally induced depolarization was observed in this FI at a radiation power up to 1270 W (Figure 9, triangles). It was proved that these polarization distortions would not be observed even at powers exceeding 2.5 kW. The obtained result makes high-purity CVD-ZnSe one of the most promising materials for creating Faraday isolators with a multikilowatt power operating at room temperature.

### 2.5. Spinel

#### 2.5.1. Production Technique

The ceramics of magnesium aluminate (MgAl_2_O_4_) and zinc aluminate (ZnAl_2_O_4_) spinels are of the greatest interest for laser applications. They have high thermal conductivity (about 18 W/m/K at 300 K [67]), microhardness (*H*_V_ about 16 [68], and 11 GPa [69], respectively), a wide range of transparency (0.2–6 and 0.3–7 μm, respectively), radiation resistance, and an isotropic structure.

An approach to the synthesis of stoichiometric MgAl_2_O_4_ nanopowders by means of hydrolysis of double magnesium-alumininate isopropoxide MgAl_2_(OPr*^i^*)_8_ with subsequent annealing of the formed hydroxides in air at a temperature of 700–900 °C was proposed in [68,70]. Double magnesium-aluminate isopropoxide has a relatively high volatility, and a particularly pure precursor of spinel nanopowders can be obtained from raw materials of technical qualification by the method of single vacuum distillation [71]. A sintering LiF 3 wt%. additive was introduced at the hydrolysis stage to ensure its uniform distribution in the powder. This evidently explains the relatively small grain growth after consolidation (the main mode was from 2.5 to 20 µm) compared to the results of the studies where lithium fluoride was introduced into spinel powder by ball milling [72]. Hot pressing of the powders was done in a graphite mold at a temperature of 1600 °C with an soaking time of 1 h. MgAl_2_O_4_ ceramics with a thickness of 1 mm had an optical transmittance of 83.7% at a wavelength of 1100 nm.

Zinc and aluminum isopropylates do not form volatile double alkoxides; therefore, we have developed a somewhat different method for the synthesis of ZnAl_2_O_4_ powder [73]. It consisted in the hydrolysis of aluminum isopropoxide with an aqueous solution of zinc formate, followed by drying and calcination of the resulting sol at a temperature of 700 °C for 20 min. Up to 6% mass of zinc fluoride was added to the initial solution as a sintering additive. The powder was consolidated into transparent ceramics by hot pressing at 1520 °C for 4 h. ZnAl_2_O_4_ ceramics with a thickness of 4 mm had an optical transmittance of about 83% at a wavelength of 1100 nm.

For doping spinels at the stage of hydrolysis, nitrates of transition metals, including rare-earth metals, were introduced. Ions of “light” elements, such as Ti, Cr, Fe, Co, and Ni have a high solubility in spinels and practically did not reduce the optical transmittance of ceramics outside their absorption lines, see, for example [73,74]. Whereas the introduction of up to 0.5% mol. rare earth oxides, including Sc and Y, led to high scattering in the spinels due to the formation of secondary phases of the corresponding aluminates [75].

#### 2.5.2. Results of the Studies

**Figure 10:**
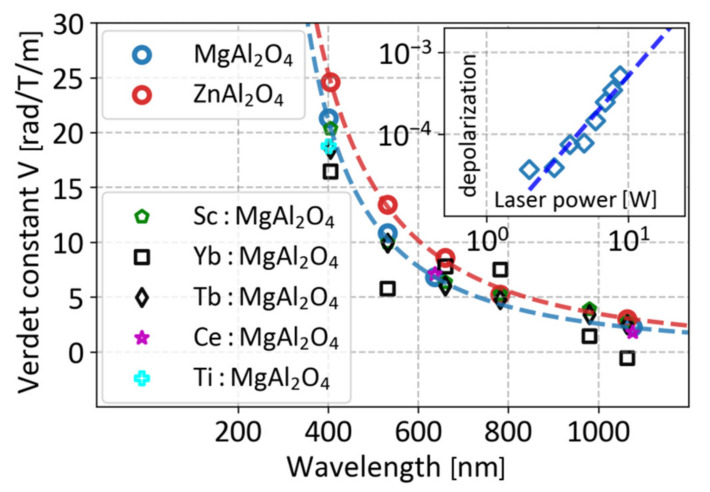
Spectral dependence of the Verdet constant in spinel samples: MgAl_2_O_4_ (red circles), ZnAl_2_O_4_ (blue circles), Sc(0.2%):MgAl_2_O_4_ (pentagons), Yb(0.5%):MgAl_2_O_4_ (squares), Tb(0.1%):MgAl_2_O_4_ (diamonds), Ce(0.5%):MgAl_2_O_4_ (asterisks), Ti(1.8%):MgAl_2_O_4_ (plus signs). Dashed curves correspond to the approximation of dispersion dependences for samples of undoped spinels. Inset: thermally induced depolarization as a function of laser radiation power in a sample of magnesium–aluminate spinel re-calculated for the length *L*_45_.

In this section we consider the results that have not been published before. We investigated the spectral dependences of the Verdet constant of magnesium-aluminate (including with various dopants such as Sc, Ti, Tb, Ce, and Yb) and zinc–aluminate spinels in the VIS-IR wavelength range (405–1064 nm), that are presented in Figure 10.

The undoped spinel demonstrates diamagnetic rotation of the plane of polarization. Its spectral dependence can be approximated by the expression (9) to an accuracy better than 3.2% with the coefficients *C* = 2.47∙10^6^ rad∙nm^2^/(T∙m) and *λ*_0_ = 208 nm. At the wavelength of 1064 nm, spinel is ~15 times inferior to TGG in terms of the Verdet constant.

As can be seen from Figure 10, doping of the spinel (with all the listed above ions except Yb) in the considered concentrations does not lead to a significant change in the Verdet constant. The deviations from the dispersion dependence, corresponding to the undoped spinel, can be explained by the measurement error same as in the case of doped TAG ceramics. More significant deviations observed for Yb:spinel can be attributed to the presence of absorption lines of the Yb^3+^ ion in the considered wavelength range, the proximity to which can significantly change the form of the dispersion dependence, as in the case of Dy_2_O_3_ ceramics [62], when doping significantly degrades the optical quality of the medium, reduces transmittance, and increases the scattering of transmitted radiation.

Thermally induced depolarization was measured in a sample of undoped magnesium—aluminate spinel. The inset in Figure 10 shows the measurement results for a 3-mm thick sample re-calculated for the length *L*_45_ in the case of a magnetic field with a strength of 2.5 T. Thermally induced depolarization is expected to provide an isolation ratio of >30 dB in such an FI with a maximal radiation power *P_max_* of ~15 W only.

Zinc-aluminate spinel also exhibits diamagnetic rotation of the plane of polarization. Its spectral dependence can be approximated by the expression (9) to an accuracy of ~10% with the values of the coefficients *C* = 3.39∙10^6^ rad∙nm^2^/(T∙m) and *λ*_0_ = 163 nm, i.e., its Verdet constant is 15–30% higher than the Verdet constant of MgAl_2_O_4_ in the investigated wavelength range.

Thus, the magneto-optical characteristics of both types of spinel are significantly inferior to those of the popular magneto-optical materials. This is the main limitation of its use in magneto-optical devices at the moment.

## 3. Laser/Active Ceramics

Yb:YAG is perhaps most often used for creating lasers with high average and peak power because of the optimal combination of laser (long lifetime and gain cross section) and material (high thermal conductivity) characteristics, as well as its low cost [76,77,78,79,80]. Works aimed at creating pulse-periodic Yb:YAG lasers with high average and peak power for scientific and industrial applications are underway is different countries: HiLASE (Dolni Brezany, the Czech Republic), Genbu (Osaka, Japan), DiPOLE (Chilton, UK) [81,82,83], and others. However, the aperture of the available single-crystal elements is insufficient to obtain the output energy of a few to tens of Joules.

Quite a number of other factors should be taken into consideration concerning the use of Yb:YAG. A serious problem arises in disk laser amplifiers: due to the high gain in the medium, the energy stored in the AE is strongly limited by the effects of amplified spontaneous emission (ASE) and parasitic generation. During cryogenic cooling, there occur both a significant undesirable decrease in the width of the gain spectrum and a shift of its center wavelength *λ*_max_. At a temperature of 80 K, the spectrum width is comparable to the shift of *λ*_max_. Therefore, for a cryogenic Yb:YAG amplifier, it is necessary either to artificially shift the lasing wavelength of the Yb:YAG generator operating at room temperature, or to cool the crystal of the Yb:YAG generator. In this case, the duration of the output pulses is limited to ~2 ps, which is too long for a number of applications. One of the options for solving these problems is to use a medium with a lower emission cross section than that of Yb:YAG to suppress the ASE effect and with a spectrum that enables operation at cryogenic temperatures. To date, the commonly used broadband media Yb:KYW, Yb:KGW, and Yb:CaF_2_ working with ultrashort pulses [84,85,86] fit this description. However, in Yb:CaF_2_, the emission cross section is too small to efficiently extract the energy stored in the active element, and the thermal conductivity of Yb:KYW and Yb:KGW, that is lower than that of Yb:YAG, limits the average output power.

Thus, to increase the output energy and efficiency of modern disk lasers it is necessary to develop and implement large-aperture AE technology, as well as to identify new active laser media with a more suitable gain spectrum and high thermal conductivity. A promising approach to solving these problems is to use optical laser ceramics. The currently available technologies make it possible to manufacture large-aperture elements tens of centimeters in diameter [87] and to create active elements from media that are hard or impossible to grow in the form of large single crystals. As was mentioned above, examples of such media are the ceramics of rare-earth-metal sesquioxides Yb:Y_2_O_3_, Yb:Lu_2_O_3_ and Yb:Sc_2_O_3_ whose thermal conductivity is higher and gain spectrum is wider than in Yb:YAG [88,89,90]. As the gain is lower than in Yb:YAG, the energy is more effectively stored in the AE. As the gain is higher than in Yb:CaF_2,_ the stored energy can be extracted more effectively. The thermal conductivity that is higher than in Yb:KYW and Yb:KGW enables broadband radiation gain at a higher average power. In addition, as will be shown below, the central wavelength and gain bandwidth of Yb:Y_2_O_3_ and Yb:Lu_2_O_3_ allow using such a cryogenic amplifier together with the available water-cooled master oscillator based on Yb:YAG crystals.

### 3.1. Yb:YAG Ceramics

In contrast to rare-earth oxides, self-propagating high-temperature synthesis (SHS) of YAG powders does not ensure production of ceramics with high optical transparency [91]. A possible reason for this is the contamination of powders with carbon [92], the removal of which requires calcination at high temperatures, leading to agglomeration of powders and a decrease in their sinterability. Consequently, we used an alternative method for their preparation—the sol-gel synthesis [93]. A method for preparing powders from a mixture of yttrium hydroxoacetate sols [94] and boehmite by spray drying [95], as well as direct sintering of compacted amorphous xerogel from a mixture of aluminum hydroxonitrate sols [96] and yttrium hydroxoacetate [97] was developed. Ceramics with high optical transparency were produced by free vacuum sintering at a temperature of 1760 °C for 2 h, followed by annealing in air at 1300 °C for 1 h. These methods can be effectively used for manufacturing active ceramic elements of disk configuration. In Russia, there are several scientific groups involved in the development and production of laser ceramics, including Yb:YAG ceramics. We also tested the ceramics manufactured at the Institute of Electrophysics of the Ural Branch of the Russian Academy of Sciences and the Ural Federal University (Yekaterinburg, Russia), according to the original method, with the nanopowders obtained by laser ablation [98].

Since Yb:YAG ceramics, as well as Yb:YAG crystals are popular and well-studied materials, we have not investigated them at room temperatures in ample detail and have not obtained significant results. Therefore, we will only give references to our works in which the laser properties of Yb:YAG ceramics were studied [99,100]. However, cryogenic cooling of active elements is known to be a significant advantage in the development of lasers with high average and high peak power, which improves both thermo-optical and laser characteristics.

Within the framework of collaborative research, we have obtained a number of samples of Yb:YAG ceramics with a diameter of 20 mm, a thickness of 1.4 mm and 5 at.% doping, manufactured at the AMRC LAB, Nanyang Technological University, Singapore. The ceramic samples were produced by the method of reaction sintering in vacuum at a temperature of 1770 °C. Details of this technology are described in [101]. Depending on the duration of sintering at a temperature of 1770 °C varying from 8 to 40 h, it is possible to obtain ceramics with an average grain size from 6 to 25 μm. Studies of three samples of different grain size showed that the behavior of their spectral characteristics on cooling is close to that observed in single crystals. Due to the large thickness of the samples, the lifetime was measured only at the liquid nitrogen temperature (when there was no re-absorption from the lower working laser level) and amounted to ~ 900 μs.

**Figure 11:**
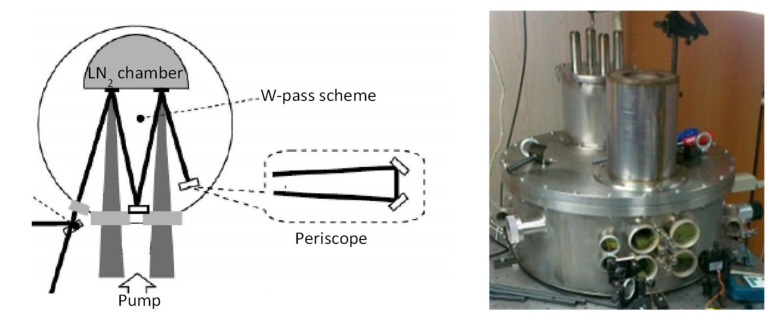
Optical scheme and a photograph of vacuum cryostat of a multipass cryogenic amplifier based on Yb:YAG ceramics.

Using the above samples, we developed a cryogenic Yb:YAG disk laser, in which the generated pulse with a duration of 70 ns and an energy of 3–5 mJ was amplified in a preamplifier to 54 mJ at a repetition rate of 200 Hz. With the use of two disk AEs made of Yb:YAG ceramics in a multipass cryogenic amplifier (Figure 11) with a filling cooling system, we obtained 233 mJ in 70 ns pulses at a repetition rate of 143 Hz with a high (20%) pump efficiency. These output parameters are the best among the currently developed cryogenic pulse-periodic laser systems based on thin disks made of Yb:YAG ceramics [102].

### 3.2. Sesquioxide Ceramics

#### 3.2.1. Manufacturing and Spectral Properties

The initial nanopowders of REE oxides were obtained by the SHS technique described in Section 2.3.1. Oxide powders were successfully synthesized on the basis of the Sc_2_O_3_ [103,104], Y_2_O_3_ [105] and Lu_2_O_3_ [106,107] matrixes doped with active REE ions, the media doped with Yb^3+^ ions being of major interest. The powders were consolidated into laser ceramics by means of free vacuum sintering and hot pressing, as well as by a specially developed microwave sintering technique. The microwave sintering was performed in the working chamber of a gyrotron complex operating in a CW mode at a frequency of 24 GHz with an automatically controlled microwave power of up to 6 kW. The compacted powders were placed in the center of a quartz crucible, covered with granular yttrium oxide for thermal isolation, and heated in a residual air atmosphere at a pressure of about 10 Pa in a microwave field to a temperature of about 1720 °C. This method was successfully used to produce (Yb_0.05_Y_0.85_La_0.1_)_2_O_3_ [108]. The laser quality was confirmed by the generation in a quasicontinuous mode. The differential efficiency with a maximum at a wavelength of 1033 nm was 25% [109,110].

**Figure 12:**
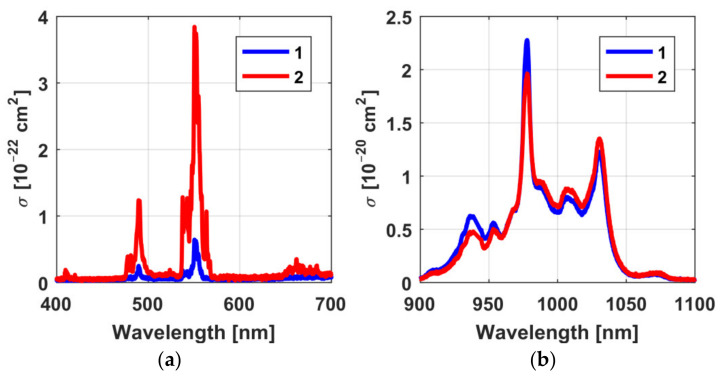
Luminescence spectra in (**a**) visible and (**b**) near-IR range of Yb:Y_2_O_3_ ceramics samples.

We also tested the Yb:Y_2_O_3_ ceramics manufactured at the Institute of Electrophysics of the of the Ural Branch of the Russian Academy of Sciences and the Ural Federal University (Yekaterinburg, Russia) [111]. Samples of the ceramics produced by these two methods were compared in the paper [112]. We attributed the difference in the generation efficiency to chemical purity of the ceramic elements that manifested itself by luminescence. Four groups of spectral lines with maxima at 409.6, 490, 550, and 662 nm were observed in the visible spectra of this material (Figure 12). The radiation in these wavelength ranges is usually associated with cooperative processes of the interaction of a Yb^3+^ ion with other REM ions (e.g., the radiation at ~409, ~550, 650–680 nm with Yb^3+^–Er^3+^, and at 490 nm with cooperative luminescence of Yb^3+^–Yb^3+^ clusters, and so on). Thus, prior to fabricating the active element, one can compare the intensity of luminescence in the visible range with the reference sample and, thus, obtain information on the spectral properties of the ceramic elements, in addition to their optical quality.

The Sc_2_O_3_ material has high thermal conductivity (18 W/m/K for an undoped material), low phonon energy, and large ground state splitting of doping ytterbium ions, which makes it a potential candidate to be used for AE lasers with high average power. 2% Yb:Sc_2_O_3_ ceramics were produced from SHS-powders containing 1 wt% of LiF sintering additive by hot pressing at 1600 °C for 1 h [104]. Despite the relatively low temperature and sintering time, secondary grain re-crystallization was initiated having bimodal distribution with mode maxima at about 10 and 30 µm. The ceramics transmittance in the near-IR range was about 78%, which is approximately 2% lower than the theoretical value. The maximum of the luminescence spectrum compared with the maximum of the Yb^3+^ ion luminescence in the YAG, Y_2_O_3_, and Lu_2_O_3_ matrices is shifted to the region of ~ 1.04 μm due to the larger splitting of the laser level. The width of the luminescence spectrum is ~10 nm, which will allow using this material to generate laser pulses with a duration of ~100 fs. The laser quality was confirmed by lasing in a quasicontinuous mode. The differential efficiency with a maximum at a wavelength of 1040 nm was 24%.

For the generation and amplification of ultrashort pulses, a laser material with a wide gain band is required. The gain band of pure sesquioxide materials is sufficient to generate and amplify pulses with a duration of ~100 fs. To obtain a wider gain band using the same materials, several active elements from different materials or solid melts can be used. For example, the ceramics with this composition (Lu_0.65_Y_0.25_La_0.05_Yb_0.05_)_2_O_3_ were manufactured by free vacuum sintering [113] at a temperature of 1750 °C. The spectral properties of solid solutions of sesquioxide ceramics were investigated in [113,114]. A number of the studied ceramics samples were also produced at the Institute of Electrophysics, Ural Branch of the Russian Academy of Sciences (Yekaterinburg, Russia) by means of solid-phase sintering of nanopowders of complex chemical composition. When some of the Y^3+^ cations were replaced by Lu^3+^ cations in the yttrium oxide matrix, the maxima of the luminescence bands of Yb^3+^ ions were shifted to the long-wavelength region of the spectrum from 1030 nm to 1032 nm. Besides, the addition of Lu_2_O_3_ leads to broadening of the gain band profile from 15 to 18 nm at half maximum for the peak in the vicinity of 1032 nm. The laser quality was confirmed by lasing on ceramic samples (Lu_0.65_Y_0.25_La_0.05_Yb_0.05_)_2_O_3_ with a differential efficiency of 32.3% and a maximal average power of 10 W.

#### 3.2.2. Studies of Thermo-Optical Characteristics

We investigated the thermo-optical properties of Y_2_O_3_, Lu_2_O_3_, and Sc_2_O_3_ ceramics samples doped with Yb^3+^ ions with concentrations of 1.8%, and 2.5%, respectively [115], manufactured by Konoshima Chemical Co. Ltd. (Kagawa, Japan), a leader in the production of commercially available laser ceramics, and a sample of a Yb:YAG single crystal with an Yb concentration of 10%. The analysis of the results showed that the Lu_2_O_3_ and Sc_2_O_3_ materials have close values of $Q_{eff},$ while for Y_2_O_3_ this value is 30% lower. Since thermally induced depolarization is proportional to the square of $Q_{eff}$/κ, this difference for the same heat release will lead to a more than twofold difference in integral depolarization degree. By comparing the values of $Q_{eff}$ and taking into account the dependence of the thermal conductivity coefficient of these materials on the concentration of Yb^3+^ ions [116] we can say that for producing AEs with a high concentration of Yb^3+^ ions, Lu_2_O_3_ ceramics is more beneficial in terms of minimization of thermally induced polarization distortions, as for the concentrations >4 at.%, thermally induced depolarization in this material will be less than in Y_2_O_3_ and Sc_2_O_3_, other conditions being equal. Contrariwise, for AEs with a low Yb^3+^ ion concentration, Sc_2_O_3_ ceramics is more beneficial, as its value of $Q_{eff}$ only 15% less, whereas the thermal conductivity coefficient increases with decreasing Yb^3+^ concentration much faster than in Y_2_O_3_. For the Yb^3+^ concentrations lower than 1 at.%, the thermally induced depolarization arising in Sc_2_O_3_ will be less than in Y_2_O_3_ or Lu_2_O_3_, other conditions being equal.

Analogously, in Ref. [115] we compared the optical powers of thermal lenses of the above ceramics. Comparison of the thermo-optical characteristic *P* singles out the Sc_2_O_3_ material, in which this value is significantly lower than in Y_2_O_3_ and Lu_2_O_3_. Like for polarization distortions, according to Equation (1), *D* is inversely proportional to the thermal conductivity coefficient, which itself is a function of the concentration of the Yb^3+^ ion. For Sc_2_O_3_, the *P/κ* value is less than for Y_2_O_3_ and Lu_2_O_3_ in the entire range of Yb^3+^ concentrations presented in Ref. [116]. The lower the thermo-optical characteristic *P*, the lower the optical power of the thermal lens introduced by the volume of the optical element is.

The thermo-optical properties of Ho:Y_2_O_3_ (0.5, 1, and 2 at.% Ho) ceramic samples manufactured at the Kotelnikov Institute of Radio Engineering and Electronics (Moscow, Russia) were investigated in the work [117]; the dependence of the thermal conductivity coefficient on the concentration of holmium ions was measured using phase-shifting interferometry [118]. The thermal conductivity coefficient of the samples under study correlated with the concentration of Ho^3+^ ions; the higher the concentration, the lower the thermal conductivity coefficient, which decreased by ~11% with a decrease in the concentration of Ho^3+^ ions from 0.5 to 2 at.%.

#### 3.2.3. Studies of Laser Properties

**Figure 13:**
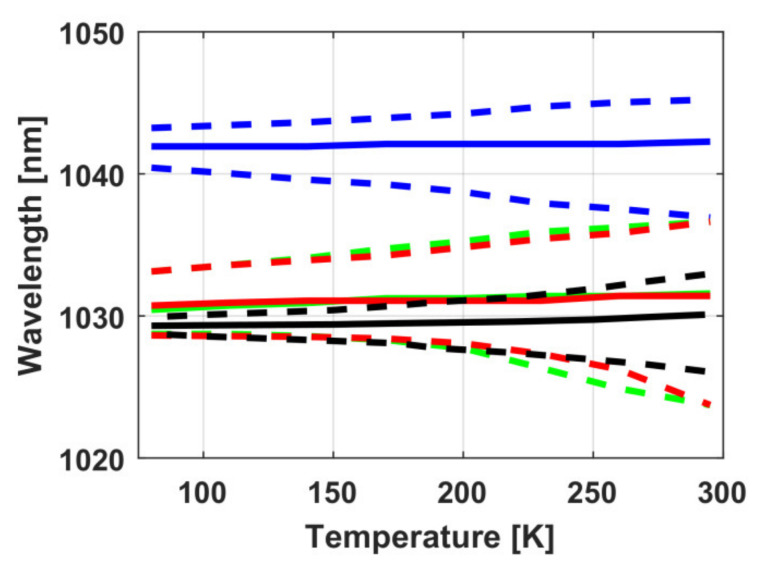
The wavelength with maximal emission cross section *λ*_max_ (solid curves) and wavelengths at which emission cross section is ½ of the maximal value (dashed curves) as a function of temperature in Yb:YAG crystal (black curves) and in Yb:Y_2_O_3_ (red curves), Yb:Lu_2_O_3_ (green curves) and Yb:Sc_2_O_3_ (blue curves) ceramics samples.

The laser properties were analyzed on the same Konoshima Chemical Co., Ltd (Kagawa, Japan). Samples [119]. The solid curves in Figure 13 depict the temperature dependence of the wavelength with maximum emission cross section *λ*_max_ in Yb:Y_2_O_3_ (red curves), Yb:Lu_2_O_3_ (green curves) and Yb:Sc_2_O_3_ (blue curves) ceramics samples, and in a Yb:YAG crystal (black curves) for comparison. The dashed curves of the corresponding color denote the values of the wavelengths at which the emission cross section is equal to half the maximal value.

The results obtained show that Yb:Y_2_O_3_ and Yb:Lu_2_O_3_ had almost the same central gain wavelength *λ*_max_ close to Yb:YAG throughout the considered temperature range. In contrast to Yb:YAG, its small shift on cooling did not play a significant role due to the wide spectrum. Actually, Yb:Y_2_O_3_ and Yb:Lu_2_O_3_ are not inferior to Yb:YAG, but they allow amplifying wider bandwidth pulses at an arbitrary given temperature.

Most interesting is that the central gain wavelength *λ*_max_ of Yb:YAG at 300 K is very close to the central gain wavelength of Yb:Y_2_O_3_ and Yb:Lu_2_O_3_ at 80 K. Thus, the signal of the master oscillator based on Yb:YAG crystals can be effectively amplified at the terminal, high-power cryogenic stages based on ceramic AEs made of Yb:Y_2_O_3_ and Yb:Lu_2_O ceramics. On the one hand, such a hybrid laser system, in comparison with a similar one made entirely on ceramic AEs, will reduce costs on the master oscillator, since today high optical quality laser ceramics is much more expensive than single crystals. On the other hand, shorter pulses can be obtained at the output of such a hybrid system than in a cryogenic Yb:YAG laser, avoiding the problem of changing the central gain wavelength on cooling. As noted above, in Yb:Sc_2_O_3_ ceramics the central wavelength is shifted to longer wavelengths relative to 1030 nm; therefore, there is currently no practical interest in using this medium.

The first multipass disk pulse-periodic amplifiers based on AEs of Yb:Y_2_O_3_ ceramics operating at room as well as at cryogenic temperature were developed at the IAP RAS [120]. Their important feature is the use of the driving signal from preamplifiers on AE made of Yb:YAG. A disk AE made of Yb:Y_2_O_3_ with a diameter of 15 mm and a thickness of 1 mm, doped with 3.5 at.% ytterbium ions was fabricated at Konoshima Chemical Co., Ltd.(Kagawa, Japan). The measurements of the transmission spectrum of the ceramics far from the resonance absorption peaks showed a high quality of the material (losses coincided to a high accuracy with the Fresnel reflection from two faces for a refractive index of *n* = 1.89 [119], which indicates that there is no absorption or scattering in the sample). The precision measurements [121] demonstrated that the nonresonant absorption at a wavelength of ~1 µm is low, ~0.01 1/cm. The ceramic sample was placed in a vacuum cryogenic chamber with a liquid nitrogen cooling system. Since the reabsorption from the lower operating level at 77 K can be neglected, the lifetime at the upper laser level can be easily measured from the fluorescence decay over time. The lifetime was 1.12 ms, which is in a good agreement with Ref. [89]. The value of the emission cross section at a wavelength of 1030 nm of about 1.3 × 10^−20^ cm^2^ was also in a good agreement with [89,119,122]. As expected, upon cooling to 77 K, the gain bandwidth at 1030 m became narrower, and the peak value increased. As a result, multipass disk pulse-periodic amplifiers based on Yb:Y_2_O_3_ ceramics AEs were developed. On cooling with liquid nitrogen, the output power was 15.8 W at a repetition rate of 11.5 kHz, a pulse duration of 0.5 ns, and a spectrum width of 1.2 nm. In the case of water cooling, the output energy was 45 mJ at a pulse repetition rate of 10 Hz, a pulse duration of 0.7 ns, and a spectrum width of 2.1 nm. According to the results of Ref. [120], the central gain wavelength of Yb:YAG at 300 K was very close to the central gain wavelength of Yb:Y_2_O_3_ at 80 K, which demonstrated effective gain of radiation from a Yb:YAG laser operating at room temperature in a cryogenic Yb:Y_2_O_3_ amplifier without loss in spectrum width and the need to match the frequencies of cascades operating at room and cryogenic temperatures. The performed research demonstrated the high potential of using Yb:Y_2_O_3_ ceramics for further scaling and constructing lasers with high peak power and high pulse energy. Increasing the average power by increasing the repetition rate using cryogenic cooling holds great promise.

### 3.3. Yb:LuAG Ceramics

An increase in the concentration of the active ion for most materials leads to a decrease in thermal conductivity due to the difference in the atomic masses of the active ion and the ion that is replaced in the material, and the resulting enhancement of thermal effects. However, in Yb^3+^ doped materials containing Lu, this effect of changing thermal conductivity is relatively insignificant. Lutetium aluminum garnet Lu_3_Al_5_O_12_ (LuAG) belongs to such materials. It possesses high values of thermal conductivity, absorption cross section, emission cross section, and mechanical strength. It can be both, grown as a single crystal and produced as a laser ceramics with high optical quality.

We studied samples of 3 at.% Yb^3+^:Lu_3_Al_5_O_12_ ceramics (see Ref. [123]) made using a new method of nanocrystalline sintering without pressure in H_2_ (NC-PL SH) medium at the School of Material Science and Engineering, Shanghai University. For the investigation of the properties of the ceramics and obtain laser generation, the sample thickness was reduced to 0.3 mm. The studied ceramic sample demonstrated a high, close to theoretical transmittance (83.3% at *λ* = 1070 nm), as well as the absorption spectra characteristic of the Yb^3+^ ion with a maximum of *λ* = 937 nm and luminescence with a maximum *λ* = 1030 nm and a half-height width of 6.5 nm. The lifetime of the laser level was 0.927 ms.

To obtain laser generation, broadband dielectric coatings were applied to the end surfaces of the AE, antireflective on one side and reflective on the other side for the wavelength range of 930 nm–1040 nm. The element was indium-soldered to a diamond heat sink cooled by a stream of water, and placed in a laser resonator. 12 V-passes of pump radiation through the disk active element were organized in the resonator, which allowed over 88% of the radiation power to be absorbed in it. The maximum differential efficiency was observed when using an output mirror with 90% reflection. The power increase in disk lasers is known to be achieved by increasing the AE and pump spot diameters. We obtained generation for two pump spot diameters (3 and 5 mm) on the AE. The differential efficiencies were 54% at pulsed pumping regardless of the spot diameter, and 41 and 39.3% at continuous pumping for 3 and 5 mm pump spot diameter, respectively. The decrease in the differential efficiency during the transition to continuous pumping can be attributed to the presence of scattering inhomogeneities in the sample bulk and to additional heat release. The efficiency can be made higher primarily due to a better fit between the pump spectrum with the maximum absorption spectrum for Yb^3+^:LuAG, as well as due to a more optimal concentration of Yb^3+^ in the disk AE [124].

The generation of 200 W laser power at continuous pumping indicates the high quality of the ceramic material under study. Therefore, we believe that the NC-PL SH method used for the manufacturing of laser ceramics is highly promising and will allow in the future producing a high-quality ceramic material for AE for lasers with multikilowatt average power.

### 3.4. Spinel

**Figure 14:**
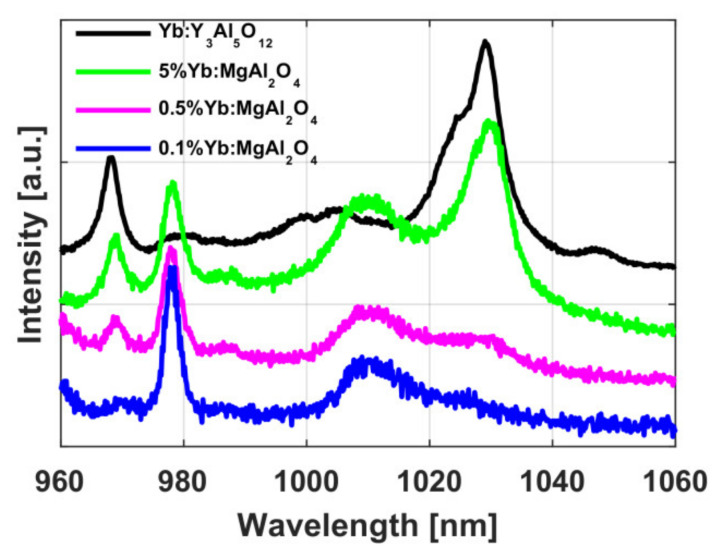
Luminescence spectra of MAS ceramics with the concentration of Yb^3+^ ions 0.1% (blue), 0.5% (magenta), and 5% (green); black curve corresponds to the luminescence spectrum of Yb:YAG crystal.

Yb:MgAl_2_O_4_ ceramics was made using the technique analogous to that described in Section 2.5.1. Ytterbium was added at the stage of hydrolysis of double magnesium-aluminate isopropoxide in the form of Yb(NO_3_)_3_ solution. The absorption and luminescence spectra of the obtained samples at a pumping wavelength of 940 nm were investigated, depending on the Yb content [75] (Figure 14). Low transmittance in highly doped samples and the peaks in the emission spectra at 1030 nm and 968 nm indicate the presence of the YbAG phase in the studied samples. Nevertheless, the 0.1% doped sample had a relatively high transmittance (more than 60% at 1 μm), its spectrum had no emission peaks at 1030 and 968 nm correlated with YbAG, there was an emission peak at 1009 nm, which we associated with the absorption and emission of the ytterbium ion in the spinel matrix (also, there were two absorption peaks at 937 and 976 nm).

The lower laser level was very close to the main level, which resulted in high re-absorption at the laser wavelength, but the quantum defect value was very small (~3.38% at *λ* = 976 nm) due to the proximity of the pump and the laser. The upper-level lifetime in 0.1% Yb ceramics was 0.7 ms. Thus, MAS optical ceramics doped with 0.1% Yb is promising for high-power applications due to its extremely low quantum defect, high thermal conductivity and mechanical properties, and possible large aperture. Therefore, we will continue to develop this trend.

## 4. Conclusions

To conclude, we would like to highlight several important points resulting from the analysis of the studies described above.

1. TGG ceramics is not inferior to TGG crystals as a medium for the FIs for high-average-power lasers, including at nitrogen temperatures. At the same time, it allows constructing large-aperture devices, thus opening up new opportunities for developing lasers with high time-average and peak power.
2. The growth technology of TAG ceramics allows introducing into its composition both, sintering additives and additional ions, which can affect the Verdet constant. However, doping of TAG ceramics with Ce, Pr, Ho, Si, Ti, and Zr in small concentrations (up to 2 at.%) does not give a significant increase in the Verdet constant. Besides, Re:TAG ceramics, that is superior to TGG in the Verdet constant, can be successfully applied in high-power FI, after further improvement of its optical properties.
3. Tb_2_O_3_, Dy_2_O_3_ and Ho_2_O_3_ sesquioxide ceramics, which are also superior to TGG in the Verdet constant, are potentially interesting throughout their transparency ranges/windows. Within the range up to ~1.4 µm, the most preferable materials are Tb_2_O_3_ and TAG (partly Dy_2_O_3_); in the range of 1.5–2.1 µm, Dy_2_O_3_ or Ho_2_O_3_ (at lower or higher λ, respectively); and in the 2.1–3.2 µm range, Ho_2_O_3_ is a leader; in the ~3.2–6 µm range, Dy_2_O_3_ is a leader; and in the range of 6–8 µm, Tb_2_O_3_ returns its leadership. Being paramagnetic, these ceramics will be of even greater interest in cryogenically cooled devices after further improvement of their optical properties.
4. Polycrystalline zinc chalcogenides (ZnSe, ZnS diamagnets) are promising MAEs for high-power FIs in their transparency windows. In particular, a high-purity CVD-ZnSe can be used for developing an FI at *λ* = 1 µm with a multikilowatt *P_max_* operating at room temperature.
5. The magneto-optical characteristics of magnesium-aluminate and zinc-aluminate spinels are significantly (more than an order of magnitude) inferior to the popular (above mentioned) MAEs. Doping with Sc, Ti, Tb, Ce, Zr and Yb ions in small concentrations (~1 at.%), like in TAG, does not lead to a significant increase in the Verdet constant. All samples demonstrate the diamagnetic rotation of the polarization plane.
6. The developed disk cryogenic laser based on Yb:YAG ceramics AE gives us ground to state that the quality of Yb:YAG ceramics is not inferior to that of Yb:YAG crystal. The achieved value of 233 mJ in 70-ns pulses at a repetition rate of 143 Hz with a high (20%) efficiency is the best among cryogenic pulse-periodic laser systems based on thin Yb:YAG ceramic disks developed at the time of this publication.
7. The analysis of the luminescence spectrum in the visible range of AE made of sesquioxide ceramics (for example, Yb:Y_2_O_3_ ceramics) shows the laser quality of ceramics in terms of chemical purity.
8. The comparison of the popular Y_2_O_3_, Lu_2_O_3_, and Sc_2_O_3_ sesquioxide ceramics doped with a Yb^3+^ ion showed that the Lu_2_O_3_ and Sc_2_O_3_ materials have similar values of thermo-optical constants $Q_{eff}$ that is 30% less in Y_2_O_3_. Therefore, the thermally induced depolarization in the latter will be ~2 times less. To fabricate AEs with a high (>4 at.%) concentration of Yb^3+^ ions, from the point of view of minimizing thermally induced polarization distortions, it is more advantageous to use Lu_2_O_3_ ceramics. Contrariwise, for AEs with a lower Yb^3+^ concentration, it is more reasonable to use Sc_2_O_3_ ceramics. The comparison of the thermo-optical constant *P* singles out the Sc_2_O_3_ material, in which this value is significantly lower than in Y_2_O_3_ and Lu_2_O_3_. (The lower *P*, the lower the optical power of the thermal lens introduced by the AE bulk is).
9. The Yb:Y_2_O_3_ and Yb:Lu_2_O_3_ ceramics are in no way inferior to the Yb:YAG material, but they allow amplifying wider-bandwidth pulses at any temperature. Most interesting is the fact that the central gain wavelength *λ*_max_ of Yb:YAG at 300 K is very close to the central gain wavelength of Yb:Y_2_O_3_ and Yb:Lu_2_O_3_ at 80 K. Thus, the signal of the master oscillator based on Yb:YAG crystals can be effectively amplified at the powerful terminal cryogenic amplification stages based on ceramic AEs made of Yb:Y_2_O_3_ and Yb:Lu_2_O_3_. In Yb:Sc_2_O_3_ ceramics, the central wavelength is shifted to a longer wavelength region therefore, in our opinion, there is no practical interest in its use at the present.
10. Despite the obvious advantages, and in some cases the irreplaceability of rare-earth oxide ceramics, their wide use in high-power laser engineering is currently limited by the complexity and weak reproducibility of their technologies. This ceramics is currently just at the beginning of its development. But there is no doubt that like the advance of the CVD-ZnSe technologies in the 1980–90 s or of the YAG ceramics at the beginning of this century, there will be progress in the production of rare-earth oxide ceramics. The scientific research and the competition will lead to a significant reduction in the cost of these materials, to improved quality and, eventually, to their widespread commercial use, hopefully in the nearest future.

## Data Availability

The authors confirm that the data supporting the findings of this study are available at the references given in the text of the article and accordingly listed in the reference list. The analyzed data are available from the corresponding author, O.V.P., upon reasonable request.
